# 

**Published:** 1886-11-13

**Authors:** 


**The Hospital.**

An Institution, Family, and Parochial Journal of Hospitals, Asylums, and all Agencies for the Care of the Sick, Criticism and News.

SATURDAY, NOVEMBER 13TH, 1886.

### The Quilter Prizes.

Mr. W. Cuthbert Quilter, M.P., has offered £50 in prizes, with the view of encouraging all engaged in hospital and asylum work to promote efficiency and economy in the management of these institutions. It is desired to give encouragement to workers everywhere, and to find expression to their views and experience for the advantage of all engaged in the administration of hospitals and asylums. There will, then, be prizes for (1) Resident Medical Officers and Superintendents, (2) for Hospital Secretaries, (3) for Lady Superintendents and Matrons, (4) for Nurses and Probationers, and (5) for Dispensers, all of whom belong to the executive staff. There will also be prizes (6) for Architects and Engineers who concern themselves with the construction and drainage arrangements of buildings designed for hospital purposes. The competition will include (*a*) the best systems of administration and control for a large hospital; (*b*) the financial arrangements, including the raising of income and the control of expenditure, with the best system of keeping the accounts; (*c*) the best system of nursing, 1, the patients, 2, selecting and training probationers, 3, planning a large hospital, so as to perfect the nursing arrangements, and to secure the maximum efficiency at the minimum of cost; (*d*) 1, the wages and relaxations of sisters and nurses; 2, their sleeping accommodation and meals; 3, their systematic and practical training; and 4, provision for sickness and old age; (*e*) the best rules for nursing a large hospital, for the guidance and control of the whole establishment, from the lady superintendent to the youngest probationer, together with the most durable and becoming uniform for a staff of hospital or asylum nurses and servants; (*f*) the best form of pharmacopœia, the best plan of a dispensary for an in- and out-patient department, and the most economical system of purchasing and dispensing drugs; (*g*) the best ground-plan, showing system of drainage, with the most complete and perfect designs for wards, day-rooms, and offices of a hospital—1, with fifty beds or under; 2, with one hundred beds and over; 3, for an asylum with five hundred patients. We propose to offer prizes from time to time in each of the above-named sections. The following are the conditions and the subject of the first competition:—

The Conditions.

1. We shall offer two Prizes of varying amounts, according to the importance attached to the subject of the competition. A cheque will be sent on the day the paper is published in which the results of each competition are given.

2. In all cases the names and addresses of the competitors must be forwarded, even when a *nom de plume* is used.

3. The answers must be addressed to "The Prizes Editor, The Hospital, Norfolk House, Norfolk Street, Strand," not later than the 15th December next, at noon. All communications must be written on one side of the paper only.

1. A Preliminary Competition.

At the outset we ask for suggestions from each of the before-mentioned classes of readers, as to the subjects in each section which they themselves consider to be the best to select for the purposes of this competition. The proposal which seems to us the most practical and in all respects the best will receive the first prize of £3, and the next best suggestion the second prize of £2.

### NOTICE TO CORRESPONDENTS.

All correspondence must be written on one side of the paper only.

We cannot undertake to return MSS. not used.

**Communications,** whether intended for insertion or for private information, must be authenticated by the names and addresses of the writers, not necessarily for publication.

**Local papers** containing reports or news paragraphs **should be marked.**

It is especially requested that early intelligence of **local events of interest,** or which it may be thought desirable to bring under notice, may be sent direct to the Editor.

Communications respecting editorial matters should be addressed to the Editor, Norfolk House, Norfolk Street, Strand, London, W.C.

### The In- and Out-patients' Hospital Diary.

(*Continued from p.* 104.)

This Diary is so arranged as to make some portion of it appear every week, and the whole in each monthly part. It has been very difficult to compile, and corrections will be very gratefully received at all times. It has been suggested that similar information about the larger Provincial and Scotch Hospitals would be useful to many readers. We should be glad to hear if this is felt to be the case.

### CHILDREN'S DISEASES—*continued from p.* 104.

| Hospitals and Terms of Admission. | Physicians, Surgeons, and Medical Officers, with Days and Hours of Attendance. |
|---|---|
| Victoria Hosp. for Children, Queen's Road, Chelsea, S.W. Age of boys, 2 to 12 years; age of girls, 2 to 16. Admission for in-patients by subscriber's letter. Accidents and urgent cases free at all times | *In-Physicians*, Drs. J. Evans, M. Tu. 2; Ridge-Jones, Tu. F. 2<br>*In-Surgeons*, Messrs. Pick, Tu. F. 9 15; Clutton, M. Th. 4.30<br>*Out-Physicians*, Drs. A. Venn, W. S. 9.30; C. Fox, M. Th. 1.30; F. D. Drewitt, Tu. F. 9; J. Philpot, M. Th. 9<br>*Out-Surgeons*, Messrs. F. Churchill, Tu. F. 10; W. Pye, M. Th. 9.30 |

### CONSUMPTION AND DISEASES OF THE CHEST.

| | |
|---|---|
| City of London Hospital for Diseases of the Chest, Victoria Park, E. By subscriber's letter available for 6 weeks. Examinations on Saturdays. | *In-Physicians*, Drs. J. Thorowgood, S.; E. Smith, M.; J. Fothergill, W.; S. West, F.; G. A. Heron, W.; V. D. Harris, Tu. Daily, 2<br>*Out-Physicians*, Drs. V. D. Harris, Th.; J. A. Ormerod, M.; E. C. Beale, Tu. F.; J. Anderson, M. F.; B. Fenwick, W. S.; A Money, W. S.; L. E. Shaw, M. Tu. Hour, 2 |
| Hospital for Consumption, Brompton, S.W. By subscriber's letter | *In-Physicians*, Drs. Thompson, M. Th.; C. Williams, M. Th.; R. Powell, Tu. F.; J. Tatham, W. S.; Thompson, W. S.; Roberts, Tu. F. Hours, 2 to 4<br>*In-Surgeon*, Mr. R. J. Godlee, F.<br>*Out-Physicians*, Drs. T. H. Green, J. M. Bruce, and J. K. Fowler, M. Th.; P. Kidd and C. Y. Biss, Tu. F.; T. D. Acland, W. S. Hour, 10 |
| Infirmary for Consumption, 26, Margaret St., Cavendish Square, W. By subscriber's letter available for 8 weeks | *Out-Physicians*, Drs. C. Torry, Jagielski, J. Barratt. Daily, 2<br>*Out-Surgeon*, Mr. F. Carr-Beard. Daily, 2 |
| North London Hospital for Consumption and Diseases of the Chest, Mount Vernon, Hampstead, N. Admission by subscriber's letter available for 6 weeks. Out-patients seen daily at Branch, 216, Tottenham Court Road, W. | *In-Physicians*, Drs. A. Evershed, Tu.; G. D. Pidcock, F.; W. Boulting, W. Hour, 10.30<br>*Out-Physicians*, Drs. J. Haward. Tu.; J. Shaw, Th.; B. O'Connor, F.; S. Taylor, M.; J. L. Cassidy, W.; J. E. Squire, S. Hour, 1 to 2. |
| Royal Hospital for Diseases of the Chest, City Rd., E.C. By Governor's letter | *In- and Out-Physicians*, Drs. Hensley, Gilbart-Smith, Finlay, White, Pringle, O. Browne, A. Davies Hobershon, E. Stewart, M. Tu. W. Th. F. 2, S. 9<br>*In- and Out-Surgeon*, Mr. Pearce-Gould, M. Tu. W. Th. F. 2, S. 9 |
| Royal National Hospital for Consumption and Diseases of the Chest, Ventnor. The London offices are at 34, Craven St., Strand, W.C. Admit only incipient cases | *In-and Out-Physicians*, Drs. J. Cockle, H. Weber, H. Port, Thorowgood, A. Davis, J. Coghill, Robertson<br>*In- and Out-Surgeons*, Messrs. Whitehead, Williamson. (Out-patients seen daily at Ventnor, 11) |

### EAR CASES.

| | |
|---|---|
| Central London Throat and Ear Hospital, Gray's Inn Road, W.C. Free, but when able, a small payment is expected | *In- and Out-Surgeons*, Dr. J. Grant; Messrs. P. S. Jakins, T. W. Jones, M. W. Th. S. 2, Tu. F. 5 |
| Charing Cross Hospital, West Strand, W.C. | *In- and Out-Surgeon*, Mr. Cantlie, F. 9 |
| Great Northern Central Hospital. See p. 16. | *In- and Out-Surgeon*, Mr. J. E. Cumberbatch |
| Guy's Hospital, St. Thomas's Street, S.E. See p. 16. | *In- and Out-Surgeon*, Mr. Purves, Tu. F. 12.30 |
| King's College Hospital, Portugal Street, W.C. See p. 16. | *In- and Out- Surgeon*, Mr. Pritchard, Th. 2.30 |

### EAR CASES.—*continued.*

| Hospitals and Terms of Admission. | Physicians, Surgeons, and Medical Officers, with Days and Hours of Attendance. |
|---|---|
| London Hospital, Whitechapel Road, E. See p. 16. | *In- and Out-Surgeons*, Dr. Woakes, Mr. T. M. Howell, S. 9<br>*In- and Out-Surgeon*, Mr. Hovell, S. 9.30, S. 3 |
| Metropolitan Ear and Throat Infirmary, 13, Howland St., W. By subscribers' letters, or by payment; free to very poor | *Out-Physicians*, Drs. Pickett, D. Nesbitt. Daily, 2.30, W. 7 |
| Middlesex Hospital, Berners Street, W. See p. 16. | *In- and Out-Surgeon*, Mr. Hensman, Tu. 9 |
| Municipal Throat and Ear Infirmary, 266, City Road, E.C. Free to necessitous; or by payment | *Out-Physicians*, Drs. G. Holmes and J. A. Hatch, M. W. F. 10 to 12, Tu. Th. 6 to 8 |
| National Hospital for the Paralysed and Epileptic, Queen Square, W.C. | *In- and Out-Surgeon*, Mr. A. Cumberbatch |
| Royal Ear Hospital, Frith Street, Soho, W. Admission by subscriber's ticket; free to the very poor | *In- and Out-Physicians*, Drs. U. Pritchard, F. Matheson, Tu. F. 9 to 12, M. S. 3 to 5 |
| St. Bartholomew's Hospital, Smithfield, E.C. See p. 16. | *In- and Out-Surgeon*, Mr. Cumberbatch, Tu. F. 2 |
| St. George's Hospital, Hyde Park, W. See p. 16. | *In- and Out-Surgeon*, Sir W. B. Dalby, Tu. 1.30 |
| St. Mary's Hospital, Paddington. See p. 16 | *In- and Out-Surgeon*, Mr. G. P. Field, W. S. 9.30 |
| St. Thomas's Hospital, Westminster Bridge Road, S.E. | *In- and Out-Surgeon*, Mr. Clutton, M. 1.30 |
| University College Hospital. See p. 16 | *In- and Out-Surgeon*, Mr. Barker, S. 9 |
| Westminster Hospital. See p. 16 | *In- and Out-Surgeon*, Mr. Barrow, Tu. F. 9 |

### EYE DISEASES.

| | |
|---|---|
| Central London Ophthalmic Hospital, Gray's Inn Rd., W.C. Admission free | *Surgeons*, Messrs. T. Archer, G. Abbott, Charnley, W. Jessop, E. Clarke, J. R. Kemp. Daily, 1 |
| Evelina Hospital for Sick Children, Southwark Bridge Road, S.E. | *Surgeon*, Dr. W. Brailey |
| Great Northern Central Hospital, Caledonian Rd., N. Free | *Surgeon*, Mr. Hutchinson, Jnr., Tu. F. 9.30 |
| Guy's Hospital, St. Thomas's Street, S.E. See p. 16 | *Surgeons*, Mr. Higgins, Dr. Brailey Tu. F. 12.30 |
| Hospital for Sick Children, 49, Gt. Ormond Street, W.C. | *Surgeon*, Mr. R. Gunn, Tu. 2 |
| King's College Hospital. See p. 16 | *Surgeon*, Mr. McHardy, M. W. Th. 1.30 |
| London Hospital, Whitechapel Road, E. See p. 16 | *Surgeons*, Messrs. W. Tay, S. 9; Eve, Tu. 9 |
| Middlesex Hospital, Berners Street, W. See p. 16 | *Surgeon*, Mr. Lang, Tu. F. 10 |
| National Hospital for the Paralysed and Epileptic, Queen's Square, W.C. | *Surgeons*, Messrs. R. Carter & M. Gunn |
| North-West London Hospital. See p. 16 | *Surgeon*, Dr. W. Collins, F. 3.30 |
| Paddington Green Children's Hospital. Free. See p. 32 | *Surgeon*, Mr. W. Jessop, Th. 9 |
| Royal Free Hospital, Gray's Inn Road, W.C. | *Surgeon*, Mr. Mackinlay, M. F. 9 |
| Royal London Ophthalmic Hospital, Blomfield Street, Moorfields, E.C. Admission free to the poor | *Surgeons*, Messrs. J. W. Hulke, G. Lawson, J. Cowper, W. Tay, J. Tweedy, Nettleship, A. Q. Silcock, Gunn, Lang. Daily, 8 to 10 |

### THE CHILDREN'S CORNER.

My little readers are evidently intent on fighting out the first quarterly contest to the bitter—or perhaps I should rather say, the friendly,—end, and, unless something goes unexpectedly wrong, there is every appearance that the result of the competition will be very close. Hetty says, "Dear Mr. Puzzle Editor, I should so like it if you would set more conundrums; they are what I like." Filomel says she likes charades, and buried cities, while Little Mary says the questions have afforded her many pleasant evenings, "they make such a lot of fun, you know," adds my little friend. The Puzzle Editor does his best to please all his little readers, but if their tastes prove so divergent, he is afraid that he himself will be "puzzled" what to do.

### OUR PASTIMES.

**Children's Quarterly Prize Competition.**

| | |
|---|---|
| Began .. .. .. .. .. | 2nd Oct., 1886 |
| Ends .. .. .. .. .. | 25th Dec., 1886 |

PRIZES.

| | |
|---|---|
| 1st .. .. .. .. | Thirty Shillings |
| 2nd .. .. .. .. | Twenty „ |
| 3rd .. .. .. .. | Ten „ |
| 4th .. .. .. .. | Five „ |

will be awarded to the four competitors, who shall have sent in, during the thirteen weeks, the largest number of correct answers to our pastimes.

**Seventh Competition.**

No. 32. I have a piece of paper measuring $4\frac{1}{2}$ inches long by 2 inches broad. How can I turn it into an exact square by making one cut only in it? (A piece of paper having the required cut should be enclosed with the reply.)

No. 33. An old wall, when pulled down, was found to have been composed of eleven thousand eleven hundred and eleven bricks. Express this number in figures.

No. 34. Buried Rivers. The following sentences contain the names of rivers. Who can discover them? "In the circular no mention is made of this," "The dog always walked behind us," "Had I genius, I should be rich," "I miss our Indian friends," "What made Xerxes lose the battle?"

No. 35. I know a word of plural number,
A foe to rest and peaceful slumber,
Yet add to this the letter "s,"
How strange the metamorphosis!
Plural is plural now no more
And sweet what bitter was before.

No. 36. Huge mountains wild and rifted rocks
Yield to *my first* a place.
Old Ocean, heaving in his bed,
*My second* doth embrace.
*My whole* to distant lands is borne,
To give to fellow man,
The power to breathe, the power to move,
Within its bounded span.

No. 37. What is that which, although it is always invisible, is never out of sight?

**Results of Fifth Competition.**

No. 21. The difference between a governess and a lady who just loses her train is simply this: the former trains the misses, and the latter misses the train!

No. 22. It is, I admit, not the simplest of matters to form a sentence limited to nine words, containing all the letters in the English language, and I am not surprised therefore that only "Little Mary" (who gives 'A quick brown fox jumps over the lazy dog"), and Tina (who gives "Jack quickly brought my favourite poodles, Xerxes and Wizard") have alone accomplished the task.

No. 23. Everybody has divined the meaning of these letters, which read

"Too wise you are, | I see you are

Too wise you be, | Too wise for me.

No. 24. Nearly all have found this out. T. K. has erred in subtracting *four* sides instead of three. The lines to be removed are as follows:

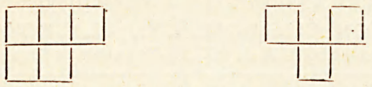

No. 25. The word of eight letters, which contains but a solitary vowel, is "Strength." Master Jimmy suggests "Brightly" as the answer, but does not Master Jimmy know that the "y" in "Brightly" is a vowel?

No. 26. "The blind beggar" was a female, and therefore the relationship was that of *sister!* I thought this question would have taxed the ingenuity of my little friends, and I was therefore agreeably surprised to find that all had found it out

Tina, a new correspondent, has attained the distinction of having answered correctly the whole of the questions in No. 5 Competition. I hope to see her example followed next week by some others of my little correspondents

All right—Tina.
Five right—Ubique.
Four right—Florrie, C. Tynget, Alsie.
Three right—T. K., Jimmy, Little Mary, Hetty Filomel.
Two right—Daisy.

Answers, having the actual name and address (to which a pseudonym may be added for publication) must have "The Children's" Coupon attached (see advertisement ages), addressed to the "PUZZLE EDITOR" THE HOSPITAL, Norfolk House, Norfolk Street Strand, W C., not later than Thursday, 18th November. Results will appear in our issue of the 27th November.

### "THE HOSPITAL" CHARITY BOX.

Our "Charity Box" is intended to afford a little practical help to the hospitals. Each week we propose a word for "anatomical dissection," and the first 150 competitors who extract the largest number of words from it receive the following prizes:—

| | |
|---|---|
| 1st .. .. .. | One Guinea. |
| 2nd .. .. .. | Half a Guinea. |
| 3rd .. .. .. | Five Shillings. |

The above Prizes will be repeated for every additional 150 competitors who may enter each week

SEVENTH COMPETITION.

Compose the largest number of words you can from

SCALPEL.

*Observe:* Proper names, abbreviations, foreign and obsolete words, and repetitions are barred.

Write only on one side of the paper, and total your words.

Append your real name and address, adding whether Mr., Mrs., or Miss, etc.

All replies, addressed "Charity Box," THE HOSPITAL, Norfolk House, Norfolk Street, W.C., must arrive by Friday, November 19th Results will appear Saturday, Nov. 27th.

*Every Competitor is required to cut out and enclose with his list the "Charity Box" Coupon* (see advertisement pages), *and a shilling entrance fee* (postal orders only).

The proceeds resulting from each "Charity Box" competition will be funded until the end of December 1886. The total sum available for distribution, and the names of the hospitals selected for donations, will appear on Saturday, January 8, 1887. The distribution of the proceeds will be at the Editor's sole discretion.

RESULTS OF FIFTH COMPETITION.

"The Hospital" Competition has resulted as follows:—

| | Score. |
|---|---|
| Miss Alice Clift, Haroldene, Vermont Road, Upper Norwood .. .. .. .. .. .. | 570—1st Prize. |
| Miss M. Williams, 47, Upper Brook Road, W. | 534—2nd Prize. |
| Mrs. A. S. Sheldon, Parkside House, Macclesfield .. .. .. .. .. .. .. .. | 533—3rd Prize. |

We shall be happy to answer in THE HOSPITAL any questions where competitors are in any doubt as to the interpretation of our Conditions.

We are glad to see that our readers are making a more general response to THE HOSPITAL "CHARITY BOX." Bearing in mind the view with which this feature was instituted, we hope that we shall receive the support of a still wider section of our readers.

In the examination of competitions, we have been somewhat hindered by the indistinct writing and general want of care in some of the papers. Others, however, are exceedingly neat and well written, the papers sent in by Miss Hadow and Miss Hannay—the latter, a young lady still at school—being especially worthy of commendation.

The prizes have been forwarded to the winners.

**Notices to Competitors.**

Miss Bedingfeld.—We allow all variations of spelling; hence we admit plurals and the second and third persons singular in verbs. We thank you for your suggestion, but fear the space at our disposal would not allow of our printing the competitions sent in by the winners of the first prize.

Mr. F. Shapley, M.R.C.S.—We much regret to hear that you are thinking of not competing again. You were very near being placed on one occasion. We are only sorry it is not possible for all to win. Competitions are not judged by any particular dictionary, and our readers are therefore quite at liberty to use any they prefer. Nuttall, Annandale, Ogilvie, and Johnson appear to be mostly employed. We hope to see you compete again.

Miss Onion.—We do not regard the second person singular as obsolete. It is still used in serious utterance and by members of the Society of Friends.

Mrs. A. S. Sheldon.—Scotch words and provincialisms are barred

Mr. C. N. Lovely.—You omitted to enclose Entrance Fee, and your competition could therefore not be considered.

